# Corticostriatal dysfunction and social interaction deficits in mice lacking the cystine/glutamate antiporter

**DOI:** 10.1038/s41380-020-0751-3

**Published:** 2020-05-04

**Authors:** Eduard Bentea, Agnès Villers, Cynthia Moore, Adam J. Funk, Sinead M. O’Donovan, Lise Verbruggen, Olaya Lara, Pauline Janssen, Laura De Pauw, Noemi B. Declerck, Erica A. K. DePasquale, Madeline J. Churchill, Hideyo Sato, Emmanuel Hermans, Lutgarde Arckens, Charles K. Meshul, Laurence Ris, Robert E. McCullumsmith, Ann Massie

**Affiliations:** 1 Neuro-Aging & Viro-Immunotherapy, Center for Neurosciences, Vrije Universiteit Brussel (VUB), Brussels, Belgium; 2 Department of Neurosciences, Research Institute for Biosciences, University of Mons, Mons, Belgium; 3 Research Services, Neurocytology Laboratory, Veterans Affairs Medical Center, Portland, OR USA; 4 Department of Neurosciences, University of Toledo College of Medicine, Toledo, OH USA; 5 Division of Biomedical Informatics, Cincinnati Children’s Hospital Medical Center, Cincinnati, OH USA; 6 Department of Biomedical Informatics, University of Cincinnati, Cincinnati, OH USA; 7 Department of Medical Technology, Faculty of Medicine, Laboratory of Biochemistry and Molecular Biology, Niigata University, Niigata, Japan; 8 Institute of Neuroscience, Université Catholique de Louvain, Brussels, Belgium; 9 Laboratory of Neuroplasticity and Neuroproteomics, and Leuven Brain Institute (LBI), KU Leuven—University of Leuven, Leuven, Belgium; 10 Department of Behavioral Neuroscience, Oregon Health and Science University, Portland, OR USA

**Keywords:** Neuroscience, Physiology, Genetics, Autism spectrum disorders

## Abstract

The astrocytic cystine/glutamate antiporter system x_c_^−^ represents an important source of extracellular glutamate in the central nervous system, with potential impact on excitatory neurotransmission. Yet, its function and importance in brain physiology remain incompletely understood. Employing slice electrophysiology and mice with a genetic deletion of the specific subunit of system x_c_^−^, xCT (xCT^−/−^ mice), we uncovered decreased neurotransmission at corticostriatal synapses. This effect was partly mitigated by replenishing extracellular glutamate levels, indicating a defect linked with decreased extracellular glutamate availability. We observed no changes in the morphology of striatal medium spiny neurons, the density of dendritic spines, or the density or ultrastructure of corticostriatal synapses, indicating that the observed functional defects are not due to morphological or structural abnormalities. By combining electron microscopy with glutamate immunogold labeling, we identified decreased intracellular glutamate density in presynaptic terminals, presynaptic mitochondria, and in dendritic spines of xCT^−/−^ mice. A proteomic and kinomic screen of the striatum of xCT^−/−^ mice revealed decreased expression of presynaptic proteins and abnormal kinase network signaling, that may contribute to the observed changes in postsynaptic responses. Finally, these corticostriatal deregulations resulted in a behavioral phenotype suggestive of autism spectrum disorder in the xCT^−/−^ mice; in tests sensitive to corticostriatal functioning we recorded increased repetitive digging behavior and decreased sociability. To conclude, our findings show that system x_c_^−^ plays a previously unrecognized role in regulating corticostriatal neurotransmission and influences social preference and repetitive behavior.

## Introduction

The astrocytic cystine/glutamate antiporter system x_c_^−^ exports glutamate in exchange for cystine in a 1:1 ratio [1]. Structurally, system x_c_^−^ is a heterodimer composed of two subunits, xCT (encoded by the gene SLC7A11) and 4F2hc, linked by a disulfide bridge. The specific subunit xCT mediates the transport activity of the complex, whereas 4F2hc is shared across various families of amino acid transporters and anchors xCT at the plasma membrane [1]. With each molecule of cystine imported by system x_c_^−^, glutamate is released in the extracellular environment. As such, system x_c_^−^ is a source of continuous, non-vesicular glutamate. In some brain regions, such as the striatum [2] and hippocampus [3], system x_c_^−^ supplies up to 60–70% of extracellular glutamate levels, revealing an important contribution to ambient glutamate levels. Given its glial localization, system x_c_^−^ delivers glutamate to the extrasynaptic compartment, where it can activate ionotropic and metabotropic glutamate receptors, and modulate excitatory signaling by fine-tuning synaptic transmission [4]. Accordingly, pharmacological and genetic studies have indicated a role of system x_c_^−^ in regulating neurotransmission at cortico-accumbens synapses [5], hippocampal CA3-CA1 synapses [6], and prefrontal cortex layer II/III pyramidal neurons [7]. The type of regulation and mechanism of action show dependency on brain circuit. System x_c_^−^ inhibits excitatory neurotransmission at the cortico-accumbens pathway via presynaptic mechanisms linked to group II metabotropic glutamate receptor activation [5]. In contrast, the inhibitory function of system x_c_^−^ at hippocampal CA3-CA1 synapses was attributed to postsynaptic mechanisms, as increased amplitude of excitatory postsynaptic potentials and expression of postsynaptic AMPA receptors were observed in xCT deficient mice [6]. Interestingly, in layer II/III pyramidal neurons, system x_c_^−^ was found to positively modulate, rather than inhibit, synaptic transmission, as decreased excitatory postsynaptic currents were obtained after system x_c_^−^ inhibition, possibly involving both pre- and postsynaptic mechanisms [7]. Supporting the fact that system x_c_^−^ participates to neurotransmission under physiological conditions, its expression modulates behavioral phenotypes, including spatial working memory [3], anxiety- and depressive-like behavior [8]. Despite its expression and robust involvement in maintaining extracellular glutamate homeostasis at the level of the striatum [2], little is known regarding the function of system x_c_^−^ in regulating neurotransmission at striatal synapses.

Psychiatric disorders, including autism spectrum disorder and obsessive-compulsive disorder (OCD) are correlated to corticostriatal deficits [9], and recent animal model studies suggest dysfunction at glutamatergic corticostriatal synapses as a causal factor for behaviors associated with autism and OCD [10, 11], that may involve neuronal [10–13] as well as glial [14] mediators. Previous findings propose a possible genetic association between variation in SLC7A11 and susceptibility to autism [15, 16]. In addition, N-acetyl-cysteine, a nonspecific system x_c_^−^ activator, reverses social interaction deficits in a rat model of autism [17] and decreases marble burying behavior in mice [18]. This is consistent with a possible role of system x_c_^−^ in establishing the excitatory–inhibitory disbalance that may occur in autism spectrum disorder [19].

In this study, we investigated whether absence of system x_c_^−^ in mice influences corticostriatal neurotransmission, the underlying mechanisms involved, and whether it impacts behavioral outcomes that are sensitive to corticostriatal function, such as social interaction and repetitive behavior.

## Materials and methods

### Animals

Adult (12–14 weeks old; 8–9 weeks for social behavior) male xCT knock-out (xCT^−/−^) mice and wild-type (xCT^+/+^) littermates are high-generation descendants of the strain described previously [20], bred in a heterozygous colony in the animal facilities of the Vrije Universiteit Brussel. The xCT^−/−^ mice were generated by targeted disruption of the START codon in exon 1 of the SLC7A11 gene and were backcrossed for more than 15 generations on a C57BL/6J background. Mice were group-housed under standardized conditions (10/14 h dark/light cycle, not reversed), with free access to food (SAFE A03, SAFE Diets) and water. Temperature in the vivarium was maintained between 20 and 24°C, and humidity between 45 and 65%. Genotypes were confirmed on ear punch DNA using the REDExtract-N-Amp Tissue PCR Kit (Sigma) as described in Supplementary information (Supplementary Fig. 1). Previous findings demonstrate that the xCT^−/−^ mice show absence of xCT mRNA [21], and are protein null across various regions of the brain [22, 23], including striatum [24, 25]. Studies were performed according to national guidelines on animal experimentation and approved by the Ethical Committee for Animal Experimentation of the Vrije Universiteit Brussel.

### Electrophysiology

**Fig. 1:**
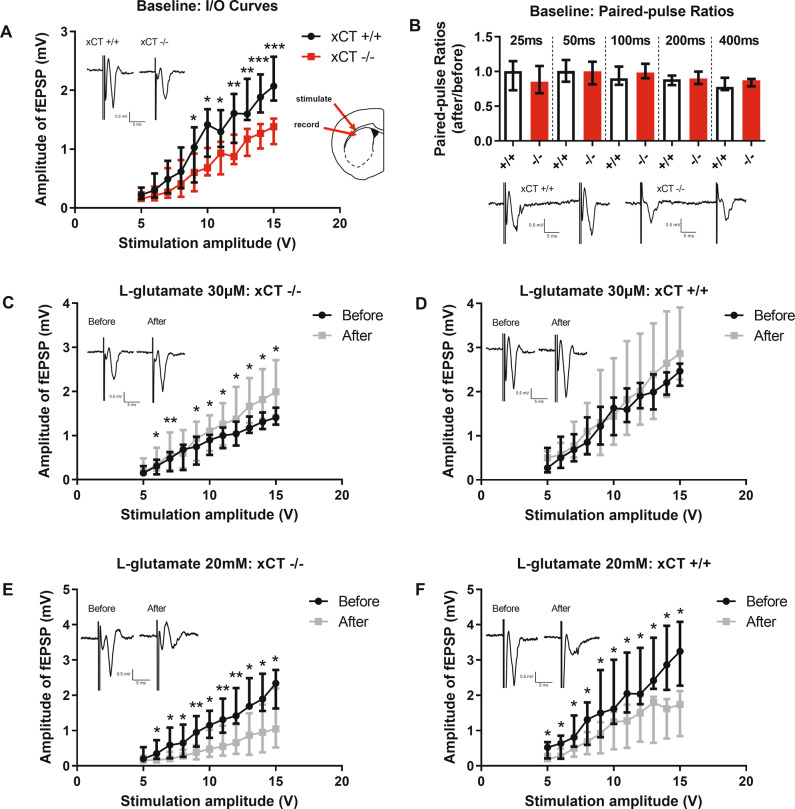
Decreased corticostriatal neurotransmission in xCT^−/−^ vs. xCT^+/+^ mice. **a** I/O curves, generated by stimulating corticostriatal fibers between 5 and 15V, revealed significantly reduced fEPSPs at the level of the dorsolateral striatum in xCT^−/−^ compared with xCT^+/+^ mice. **b** No differences between genotypes were observed in paired-pulse response ratios, when the two consecutive pulses were delivered at intervals ranging from 25 to 400 ms. Bath application of 30 µM L-glutamate for 2 h significantly increased the responses measured in xCT^−/−^ slices (**c**), without affecting responses in xCT^+/+^ slices (**d**). **e, f** Desensitization of glutamate receptors after exposure to high concentration of L-glutamate. Input/output curves, generated by stimulating corticostriatal fibers between 5 and 15V, revealed significantly reduced fEPSPs at the level of the dorsolateral striatum after bath incubation with 20 mM L-glutamate for 15 min, in both xCT^−/−^ (**e**) and xCT^+/+^ (**f**) slices. A total number of 15 slices from *n* = 8 xCT^+/+^ mice and 16 slices from *n* = 8 xCT^−/−^ mice were analyzed. All slices were tested for baseline I/O curves and paired-pulse ratios. After baseline recordings, half of the slices were incubated with 30 µM L-glutamate for 2 h followed by 20 mM L-glutamate for 15 min (*n* = 8–9 slices/genotype). Data are presented as median ± interquartile range and analyzed using a two-tailed Mann–Whitney *t* test (**a**, **b**) or a two-tailed Wilcoxon matched-pairs signed rank test (**c–f**) *t* test. *p < 0.05, **p < 0.01, ***p < 0.001, vs. xCT^−/−^ (in **a**) or vs. “before” at each corresponding voltage (in **c**–**f**). Inserts indicate example of trace fEPSPs obtained at maximum stimulation (15 V) in xCT^−/−^ and xCT^+/+^ slices under baseline conditions (**a**), and after bath application of 30 µM L-glutamate (**c, d**) or 20 mM L-glutamate (**e, f**). Inserts depicted for the paired-pulse ratios (**b**) reflect responses obtained using a 25 ms interval between pulses.

Electrophysiology was performed as described [26], with minor modifications. Brains were placed in 4 °C artificial cerebrospinal fluid (aCSF) solution (124 mM NaCl, 4.4 mM KCl, 26 mM NaHCO_3_, 1 mM NaH_2_PO_4_, 2.5 mM CaCl_2_, 1.3 mM MgSO_4_, 10 mM D-glucose) and bubbled with a mixture of 95% O_2_ and 5% CO_2_. A thick section encompassing corticostriatal fibers was cut at a 30° angle, from which 400 µm slices were cut in aCSF [27]. Slices were transferred to a recording chamber and allowed to recover for 1.5 h at interface. Recordings were made in an interface chamber (Tissue Slice Recording Chamber dual well 21051-00, FST, Canada) at 28 °C, under constant perfusion with oxygenated aCSF (1 mL/min). A bipolar twisted nickel–chrome stimulating electrode, 50 μm in diameter, was placed at the border of the corpus callosum in close proximity to the recording electrode, located in the dorsolateral region of the striatum (Fig. 1a). Extracellular field excitatory postsynaptic potentials (fEPSPs) were evoked using biphasic constant-voltage pulses (0.08 ms for each pulse) and recorded with a low resistance (2–5 MΩ) glass microelectrode filled with aCSF. Baseline input/output (I/O) curves were determined by varying the stimulus intensity and determining the amplitude of the fEPSP. Paired-pulse response ratios were obtained by delivering two stimuli with an intensity to induce 50% of the maximal response, at various intervals. Following acquisition of baseline responses, the perfusion solution was switched to 30 µM L-glutamate. Stimuli were delivered every minute using a stimulus intensity to induce 50% of the maximal response, for a total duration of 2 h. The amplitude of the fEPSP was measured on the average of four consecutive responses. At the end of the 2 h incubation, I/O curves were derived as described above.

### Golgi–Cox

Golgi–Cox staining was performed using the FD Rapid GolgiStain kit (FD Neurotechnologies Inc., USA) [28]. Neurons with clearly visible Golgi–Cox staining were selected from the dorsolateral striatum of xCT^−/−^ mice and xCT^+/+^ littermate controls. Neurons were imaged using a bright field microscope (Zeiss Axio Imager Z.1) connected to an AxioCam MRc5 camera, using Zen2 (Blue edition, version 2.0.0.0; all from Carl Zeiss Microscopy GmBH). The following criteria were utilized for inclusion of striatal medium spiny neurons (MSNs) based on their typical somato-dendritic morphology and quality of staining: (1) neuron stained in its entirety, (2) with a soma that is round or ovoid with a diameter between 11 and 20 µm, (3) which contains at least three primary dendrites, and (4) demonstrates low dendritic spine density on primary branches that increases from the second and third order branches onward [29]. Neurons were manually traced on flattened Z-stack images (0.5 µm/stack) loaded as individual image layers in GNU Image Manipulation Program (version 2.10.6), and their morphology evaluated. The following parameters were derived on the obtained traces in a random manner by an experimenter blind to the experimental conditions: soma area, soma diameter, total dendritic length, the number of primary dendrites, and the length of the longest dendrite. The complexity of the dendritic tree was determined by Sholl analysis, counting the number of intersections of the dendritic tree with concentric circles placed at increasing diameters in steps of 10 µm. Spine density was evaluated on dendritic segments between 12 and 28 µm in length, located at a distance of 60 to 90 µm from the soma. Neurons were imaged at ×40 magnification for measurement of neuronal morphology, and at ×100 magnification for dendritic spine counts. Image analyses were performed using ImageJ software (National Institutes of Health, USA).

### Electron microscopy

Electron microscopy (EM) was performed as previously described [30–32]. By using an antibody against VGLUT1 as specific marker of cortical input [33], we investigated the morphology of corticostriatal synapses after loss of xCT. Slices containing the rostral dorsolateral striatum (1.0 mm anterior to bregma), a region that receives a significant input from the motor cortex [34], were processed for VGLUT1 pre-embedding 3,3′-diaminobenzidine (DAB) immunolabeling using a rabbit VGLUT1 antibody (1:1000; Synaptic Systems; cat. #135 303) and a microwave procedure (Pelco BioWave, Ted Pella). Next, the tissue was flat-embedded in epoxy, the dorsolateral striatum manually dissected and thin sectioned (60 nm). Post-embedding glutamate immunogold labeling was performed using a rabbit glutamate antibody (1:250; Sigma; cat. #G6642) and a goat anti-rabbit IgG tagged with 12 nm gold particles (1:50; Jackson ImmunoResearch; cat. #111-205-144). Incubation of the antibody with 5 mM glutamate resulted in a near-complete loss of immunogold labeling, showing the specificity of the glutamate labeling (Supplementary Fig. 2). Photographs of VGLUT1-labeled terminals were taken at a final magnification of ×40.000 using a digital camera (AMT, Danvers, MA, USA). Terminals were analyzed if they demonstrated DAB-labeled synaptic vesicles and a clearly defined asymmetrical synaptic contact with an underlying dendritic spine (defined by its characteristic shape and absence of mitochondria) or dendritic shaft. The number of immunogold particles located inside the nerve terminal (vesicular and cytoplasmic pools are combined in the final analysis; the cytoplasmic pool accounts for <10% of the entire nerve terminal pool of glutamate [35, 36]), and those associated with mitochondria were counted. For calculating the glutamate density in presynaptic terminals, the mitochondrial pool was subtracted from the total nerve terminal pool. Background labeling within glial cell processes (ten immunogold-labeled particles/μm^2^) was subtracted from the density of presynaptic and dendritic spine immunogold-labeled glutamate within the nerve terminals and postsynaptic labeling within spines [36]. To investigate whether xCT deletion led to a global change in the number of spines or glutamatergic synapses, we quantified the total number of spines and asymmetric synapses per field of view (14 μm^2^) made by VGLUT1-labeled nerve terminals, and averaged these values over all photographs taken of each mouse. Other ultrastructural parameters that were quantified included: the percentage of axospinous vs. axodendritic contacts; the width of the synaptic cleft; the area and diameter of the presynaptic terminals; the area and diameter of spine heads; the percentage of terminals containing mitochondria; the area of mitochondria and the density of mitochondria per terminal; the percentage of synapses with a perforated postsynaptic density (PSD; a synapse was considered to show a perforation if the corresponding PSD was discontinued for a distance of at least 50 nm [35]); and measures of PSD size including length, thickness, maximum thickness, and area [37, 38]. The width of the synaptic cleft was calculated from an average of three measures of distance between pre- and postsynaptic membranes [39]. Nerve terminal and spine diameters were calculated by measuring the distance between the widest points of the terminal or spine head parallel to the PSD [40, 41]. Image analyses were performed using ImageJ software in a random manner by an experimenter blind to experimental conditions.

### Proteomics

Samples were prepared for proteomic analyses as described previously [42]. Nano liquid chromatography coupled to electrospray tandem mass spectrometry was performed on a 5600+ QTOF mass spectrometer (Sciex, Toronto, ON, Canada) interfaced to an Eksigent (Dublin, CA, USA) nanoLC ultra nanoflow system [43]. For additional details on the method and analyses, see Supplementary information.

### Kinomics

Profiling of the activity of serine–threonine kinases was performed using the PamStation12 microarray (PamGene International), as described previously [44–46]. Samples were run in duplicate using two separate chips, and the results averaged across the two arrays. The identified kinases driving the changes in peptide phosphorylation were integrated in a global network model that includes kinase interaction pathways. To investigate the role of extracellular signal-regulated kinase (ERK) in regulating the kinase network in xCT^−/−^ and xCT^+/+^ mice, each sample was evaluated with the kinome array as described above, in the presence or absence of a specific ERK1/2 inhibitor (FR 180204; Tocris 3706), added at a concentration of 5 µM [47]. For additional details on the method and analyses, see Supplementary information.

### Behavioral analyses

Behavioral tests were performed between 9:00 a.m. and 5:00 p.m. with alternate testing of xCT^−/−^ and xCT^+/+^ mice to ensure evaluation of both genotypes during the same time of day. The spontaneous grooming test, marble burying test, and social interaction tests (reciprocal social interaction and three-chambered social preference test) were performed on independent cohorts of mice. For the mice subjected to social interaction tests, the sequence of behavioral tests was the reciprocal social interaction first, followed by the three-chambered social preference test. The behavioral results were analyzed in a random manner by a researcher blind to experimental conditions.

#### Grooming behavior

Analysis of self-grooming was performed as described previously [48]. Mice were video-taped for 30 min and their behavior analyzed on the recorded video files.

#### Marble burying

Fifteen black marbles (15 mm in diameter) were placed on top of wood chip bedding (3 cm deep) in a 3 × 5 arrangement. Mice were individually placed in the cage and video-recorded from above for 15 min. A marble was considered to be buried when more than two-thirds of their diameter was covered with bedding [49]. Motor function of the mice was monitored and analyzed on the acquired video files using ToxTrac [50].

#### Reciprocal social interaction

Pairs of male, genotype-matched and socially naive (i.e., non-littermate) mice were placed in a new clean cage and allowed to interact in an unrestricted manner for 10 min. At the end of the trial, various measures of social interaction are evaluated on the recorded video files, including nose-to-nose sniffing, nose-to-body sniffing, nose-to-anogenital sniffing, following, pushing past each other with physical contact, crawling over and under each other with physical contact, chasing, mounting, and allogrooming [51].

#### Three-chambered social preference

The three-chambered social preference test was performed in a three-chambered box made of clear polycarbonate [52]. The test mice were habituated to the center chamber for 10 min, followed by 10 min with access to all three empty chambers. Then, the mice were briefly confined to the center chamber, while a novel inanimate object (an inverted wire cup) is placed in one of the side chambers, and a socially naive novel mouse is placed in an identical wire cup located in the other side chamber. Next, the test mice are allowed access to all three chambers for a 10 min trial, which is video-recorded. The time spent interacting with the novel mouse, the time spent in each chamber, and the number of entries performed in each chamber were measured on the recorded video files. As socially naive novel mice we employed 8-week old 129/SvImJ adult male mice that were maintained socially naive to the experimental group by housing them in a different room, and that were habituated to the inverted wire cup.

### Sample size estimation

Given the context-sensitive and varying role of system x_c_^−^ on regulating synaptic transmission, we did not assume that neurotransmission at corticostriatal synapses occurs differently in the xCT^−/−^ mice (and the direction of which it may occur) or that it would be unchanged. As we are the first to report the effect of system x_c_^−^ on corticostriatal neurotransmission, previous reports of expected effect size could not be used as a guide for sample size estimation. The sample size we considered fits in the range of comparable exploratory studies performed using a similar electrophysiology set-up as employed in the present study [53]. Using the change in fEPSPs as a starting point, and published effect sizes in genetic models with corticostriatal dysfunction [10, 13], we estimated the sample size required to test for deficits in behavioral tests sensitive to changes in corticostriatal neurotransmission, using G*Power 3.1.9.2 (difference between two independent means, two-tailed, *α* set at 0.05, the desired power 1-β set at 0.8). In case of the three-chambered test, a sequential stopping rule was applied [54]. As such, the sample size employed was lower than the initial estimated sample size due to exposing robust effects, thereby reducing the number of animals. For all other analyses, sample sizes were chosen based on typical values from previous studies using similar techniques. No animals or samples have been excluded from our analyses.

### Statistical analyses

Data are presented as median ± interquartile range for nonparametric statistical analyses, and mean ± standard error of the mean for parametric statistical analyses. Nonparametric statistics were performed for all comparisons in which at least one group demonstrated non-normal distribution, as evaluated using the D’Agostino and Pearson omnibus normality test, or where the groups demonstrated unequal variance, as evaluated using the Bartlett’s test. Statistical analyses were performed using GraphPad Prism 6.1 software. Statistical tests are indicated in the respective figure legend. The *α* value was set at 0.05.

## Results

### Decreased corticostriatal neurotransmission in xCT^−/−^ mice

xCT^−/−^ mice showed significantly decreased fEPSPs starting from 9 V stimulation amplitude, with the two I/O curves progressively diverging as the stimulation amplitude increased (Fig. 1a). No difference was observed in the paired-pulse ratios between genotypes (Fig. 1b), indicating absence of changes in short-term synaptic plasticity. Genetic deletion of xCT results in decreased striatal extracellular glutamate levels [2], possibly inducing decreased fEPSPs. Indeed, bath application of 30 µM glutamate led to significantly increased corticostriatal responses in xCT^−/−^ slices, with the two I/O curves diverging as the current stimulation increased (Fig. 1c). Perfusion with 30 µM glutamate had no significant effect on I/O curves derived from xCT^+/+^ slices (Fig. 1d). At the end of the protocol, slices were exposed to a high L-glutamate concentration (20 mM) for 15 min to confirm reproducible perfusion of the drugs in the recording chamber of both genotypes (Fig. 1e, f).

### Normal spine and synapse density, and neuronal morphology in the dorsolateral striatum of xCT^−/−^ mice

**Fig. 2:**
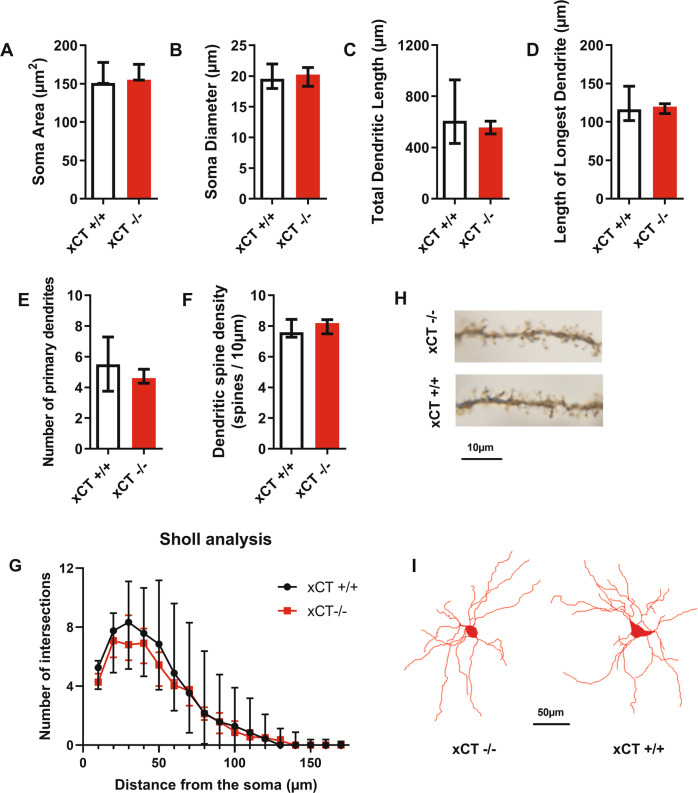
Intact dendritic spine density and morphology of MSNs in the dorsolateral striatum of xCT^−/−^ mice. Genetic deletion of xCT did not influence the area (**a**) or diameter (**b**) of MSN soma. No differences were observed between xCT^−/−^ and xCT^+/+^ mice in the total dendritic length (**c**), the length of the longest dendrite (**d**), or the number of primary dendrites (**e**). Genetic deletion of xCT did not affect the density of dendritic spines (**f**), or the dendritic arborization as assessed by Sholl analysis (**g**). High-power magnification photomicrographs (×100) of dendritic spines from xCT^−/−^ and xCT^+/+^ mice (**h**). Representative traces of MSNs stained in the dorsolateral striatum of xCT^−/−^ and xCT^+/+^ mice (**i**). Neurons with clearly visible Golgi–Cox staining were selected from the dorsolateral striatum of *n* = 4 xCT^−/−^ mice and *n* = 4 xCT^+/+^ littermate controls. Morphological analyses were obtained from an average of 10.25 ± 0.47 neurons per mouse (for xCT^−/−^) and 9.25 ± 1.37 neurons per mouse (for xCT^+/+^), for a total of 41 neurons from xCT^−/−^ mice and 37 neurons from xCT^+/+^ mice. Dendritic spine density was measured on an average of 2.06 ± 0.11 segments per neuron from 35 neurons (for xCT^−/−^) and 2.43 ± 0.17 segments per neuron from 32 neurons (for xCT^+/+^), for a total of 72 segments counted for xCT^−/−^ mice and 80 segments counted for xCT^+/+^ mice. Data are presented as median ± interquartile range and analyzed using a two-tailed Mann–Whitney *t* test.

**Fig. 3:**
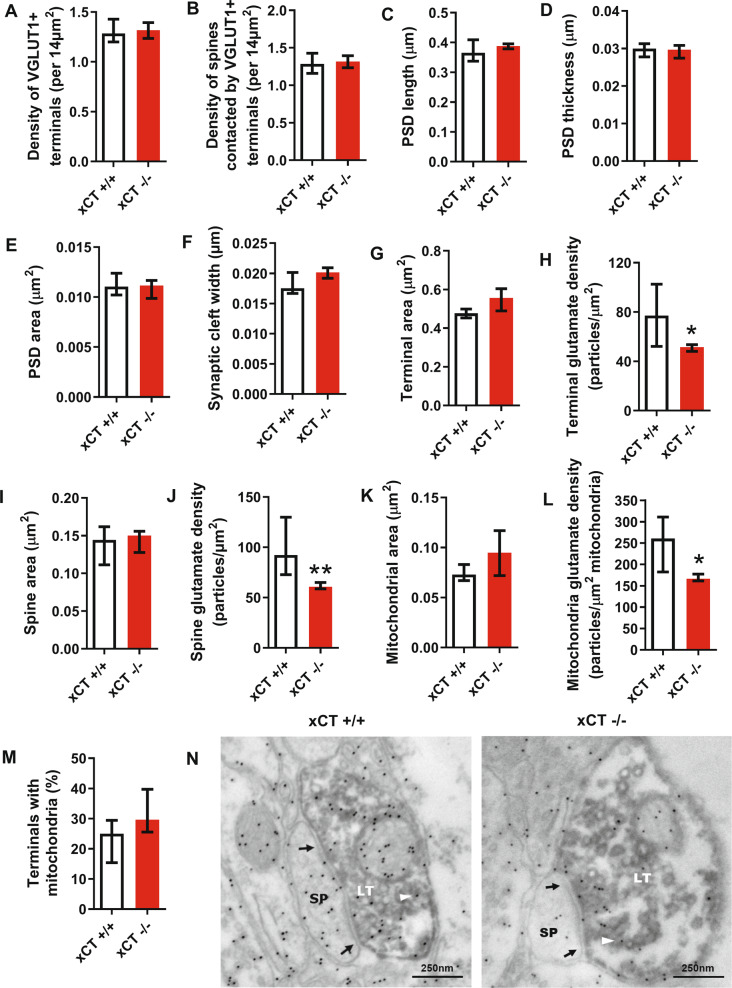
Ultrastructure of corticostriatal synapses and glutamate immunogold labeling in xCT^−/−^ vs. xCT^+/+^ mice. Genetic deletion of xCT did not affect the density of VGLUT1^+^ presynaptic terminals making an asymmetric synaptic contact (**a**), or the density of dendritic spines receiving VGLUT1^+^ synaptic input (**b**) per field of view (14 µm^2^). The total number of spines and asymmetric synapses per field of view made by VGLUT1-labeled nerve terminals were quantified and averaged over all photographs taken of each mouse. The total number of fields of view analyzed was 145 for xCT^+/+^ mice (average of 20.71 ± 2.44 fields of view/mouse) and 100 for xCT^−/−^ mice (average of 20.00 ± 3.62 fields of view/mouse). Genetic deletion of xCT did not influence the length (**c**), thickness (**d**), maximum thickness (data not shown), or total area (**e**) of the PSD at VGLUT1^+^ corticostriatal synapses. Similarly, loss of xCT did not impact the average synaptic cleft width (**f**), area of presynaptic terminals (**g**), dendritic spines (**i**), or presynaptic mitochondria (**k**), and had no effect on the % of terminals with mitochondria (**m**). However, absence of xCT led to a significant depletion of the density of glutamate immunogold labeling from presynaptic terminals (**h**), dendritic spines (**j**), and presynaptic mitochondria (**l**). A total number of 182 VGLUT1-labeled terminals from *n* = 7 xCT^+/+^ mice (average of 26.00 ± 3.32 terminals/mouse) and 129 VGLUT1-labeled terminals from *n* = 5 xCT^−/−^ mice (average of 25.80 ± 4.60 terminals/mouse) were analyzed. Data are presented as median ± interquartile range and analyzed using a two-tailed Mann–Whitney *t* test. *p < 0.05, **p < 0.01. Representative electron micrographs of the glutamate immunogold labeling at VGLUT1^+^ axospinous synapses in the dorsolateral striatum of xCT^+/+^ and xCT^−/−^ mice (PSD defined by arrows, glutamate immunogold labeling indicated by arrowhead) (**n**). LT labeled terminal, PSD postsynaptic density, SP spine.

Using Golgi–Cox staining we could not identify changes in the morphology of MSNs located in the dorsolateral striatum of xCT^−/−^ mice, compared with xCT^+/+^ controls (Fig. 2a–e). In addition, no changes were revealed in the number of intersections of the dendritic tree at different distances from the cell body (Fig. 2g, i). The density of dendritic spines measured on segments of branches of MSNs was unchanged between the genotypes (Fig. 2f, h). EM confirmed these findings, as the density of corticostriatal terminals and dendritic spines contacted by VGLUT1^+^ terminals at the level of the dorsolateral striatum, per field of view of acquired electron micrographs, was similar in xCT^−/−^ and xCT^+/+^ mice (Fig. 3a, b).

### Normal synaptic ultrastructure at corticostriatal synapses in xCT^−/−^ mice

xCT^−/−^ and xCT^+/+^ mice have a similarly sized PSD at corticostriatal synapses (Fig. 3c–e). No significant differences were detected between xCT^−/−^ and xCT^+/+^ mice in the synaptic cleft width (Fig. 3f), VGLUT1^+^ terminal area (Fig. 3g), head diameter (data not shown), dendritic spine area (Fig. 3i), dendritic spine head diameter (data not shown), mitochondrial area (Fig. 3k), percentage of VGLUT1^+^ terminals containing mitochondria (Fig. 3m), or percentage of synapses with a perforated PSD (data not shown).

### Decreased glutamate density at corticostriatal synapses in xCT^−/−^ mice

Genetic xCT deletion induced decreased glutamate labeling across different synaptic compartments, including VGLUT1^+^ presynaptic terminals (Fig. 3h), dendritic spines contacted by VGLUT1^+^ terminals (Fig. 3j), and mitochondria located in VGLUT1^+^ presynaptic terminals (Fig. 3l).

### Proteome changes in the striatum of xCT^−/−^ mice

**Fig. 4:**
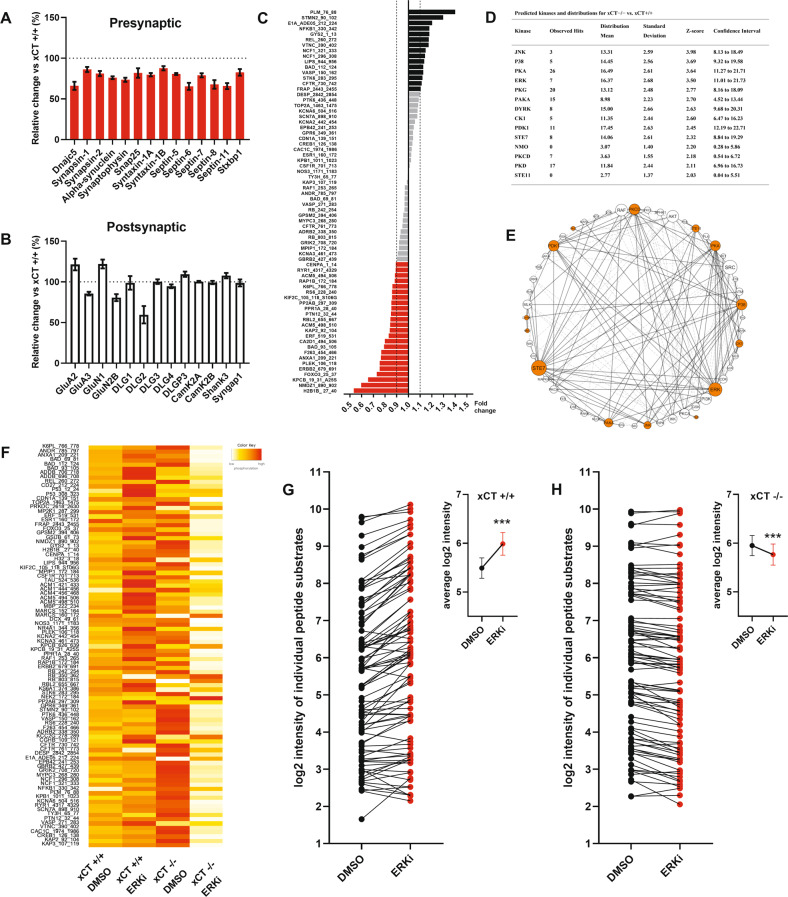
Proteomic and kinomic analysis of xCT^−/−^ vs. xCT^+/+^ mice. **a**, **b** Altered synaptic proteome in the striatum of xCT^−/−^ vs. xCT^+/+^ mice. Discovery-based data-dependent acquisition (DDA) shotgun proteomics revealed changes in expression of presynaptic and postsynaptic proteins in the striatum of xCT^−/−^ mice (pool of *n* = 10 biological replicates) compared with xCT^+/+^ littermates (pool of *n* = 9 biological replicates). Samples were run in technical triplicate. Relative quantification of peptide intensity levels indicated overall lower levels of presynaptic proteins in xCT^−/−^ mice (**a**), while the pattern of changes in postsynaptic proteins was more variable (**b**). The intensity of each protein was derived based on the geomean of the common set of peptide fragments identified across the samples analyzed normalized to total protein levels. Plotted are the relative changes in protein intensity in each of the three technical replicates of the pooled xCT^−/−^ sample when compared with the average of the three technical replicates of the pooled xCT^+/+^ sample set as 100%. **c**–**e** Kinase network dysregulation in xCT^−/−^ vs. xCT^+/+^ mice. Kinome array analysis led to the identification of serine–threonine kinases predicted to be differentially active in striatal tissues of xCT^−/−^ mice (pool of = 10 biological replicates) compared with xCT^+/+^ mice (pool of *n* = 9 biological replicates). **c** Waterfall plot showing changes in degree of phosphorylation at reporter peptides in the striatum of xCT^−/−^ vs. xCT^+/+^ mice. Peptides with increased (FC > 1.10) or decreased phosphorylation (FC < 0.90) are highlighted in black and red, respectively. Using the substrates identified in **c** as input, we performed kinase random sampling analysis to identify the upstream kinases that are most likely driving these effects. This led to the identification of 14 different serine/threonine protein kinase families (**d**) predicted to be differentially active in xCT^−/−^ vs. xCT^+/+^ mice. Based on the kinases identified in the statistical model, a kinase network model (**e**) was constructed by growing the kinome array hits with kinase interacting partners as identified using STRING. In this model, the kinome array hits from **d** are colored in orange, while indirect hits obtained after growing the network in STRING are depicted with white circles. The size of the circles corresponds to the number of interactions, with larger circles having more interactions. Thick lines represent interactions with a kinome array direct hit, while dashed lines represent interactions made between kinome array indirect hits. Kinase network model generated using Cytoscape ver. 3.6.1. **f**–**h** ERK as a node of kinase network dysregulation in xCT^−/−^ mice. ERK inhibition has contrasting effects on kinase network activity in xCT^−/−^ and xCT^+/+^ samples. **f** Global phosphorylation heatmap, depicting the relative signal intensity at each reporter peptide for the conditions indicated. Striatal lysates of xCT^−/−^ and xCT^+/+^ mice were incubated either with 5 µM FR 180204, a specific ERK1/2 inhibitor (ERKi), or with dimethyl sulfoxide (DMSO) as control. For ease of clarity, the heatmap is normalized per row to highlight relative changes at each individual peptide between the groups. Orange-red indicates high levels of phosphorylation and yellow-white indicates low levels of phosphorylation. Global phosphorylation plots, showing changes in degree of phosphorylation at each reporter peptide, as well as the average phosphorylation values (inset), when comparing ERKi to DMSO in xCT^+/+^ mice (**g**), and ERKi to DMSO in xCT^−/−^ mice (**h**). Data in **g** and **h** are presented as mean ± standard error of the mean and analyzed using a two-tailed paired *t* test. ***p < 0.001.

Using data-dependent acquisition (DDA) shotgun proteomics, we identified a small but consistent decrease in a large number of presynaptic proteins in the striatum of xCT^−/−^ mice (Fig. 4a). Fewer changes were observed in postsynaptic proteins, and they tended to follow a more variable pattern (Fig. 4b). By running proteomics in data-independent acquisition mode, and performing western blot using the same samples analyzed for proteomics, we identified a similar decrease in synaptophysin expression in xCT^−/−^ samples, as observed during DDA (Supplementary Fig. 3 and Supplementary Table 1).

### Kinome changes in the striatum of xCT^−/−^ mice

After excluding peptides that were undetectable, did not increase in signal linearly with exposure time in the post-wash phase or were nonspecific (Supplementary Fig. 4), 70 out of 144 substrates on the array were included in the final analysis. Using a fold change cutoff of ±10% [45, 55] for peptide selection, we identified 39 peptides that were changed in degree of phosphorylation in xCT^−/−^ vs. xCT^+/+^ striatal samples (Fig. 4c). Using these substrates as input, we performed kinase random sampling analysis to identify the upstream kinases that are most likely driving these effects [44, 45]. This led to the identification of 14 different serine/threonine protein kinase families predicted to be differentially active in xCT^−/−^ vs. xCT^+/+^ mice (Fig. 4d). A kinase network model that would better reflect kinase network dysregulations characteristic of xCT^−/−^ mice, was generated by connecting our initial kinase hits from the kinome array with kinase families to which they are known to interact with, using the STRING database. In addition, as kinase networks may be amplified depending on the number of interactions, we weighted the network by the number of connections made by each individual kinase family. The extended kinase network obtained, with the associated kinase interactions, is depicted in Fig. 4e. This analysis identified STE7, ERK, protein kinase A, and p38 as potent nodes of kinase network dysregulation in the striatum of xCT^−/−^ mice.

Based on the profile of the ERK-sensitive peptide curves on the kinome array (Supplementary Fig. 5A), we predicted a decrease in ERK activity in xCT^−/−^ mice. Accordingly, western blot analysis of the same samples submitted for kinomic analysis, revealed a decrease in both p-ERK1/ERK1 and p-ERK2/ERK2 ratios in the xCT^−/−^ mice (Supplementary Fig. 5B). In addition, phosphorylation of myelin basic protein at residue Thr229 (corresponding to residue Thr232 in the human sequence), a known phosphorylation target of ERK1/2 [56], was decreased by ~44% in our xCT^−/−^ proteomics samples compared with xCT^+/+^ controls (data not shown). Consistent with our findings of ERK as a possible node of kinase dysregulation in xCT^−/−^ mice, application of FR 180204, a specific ERK1/2 inhibitor [47], had contrasting effects on the kinase network activity in xCT^−/−^ and xCT^+/+^ samples, while in the xCT^+/+^ mice, it led to a general increase in phosphorylation, in xCT^−/−^ mice it reduced global levels of phosphorylation (Fig. 4f–h).

### Increased marble burying and social interaction deficits in xCT^−/−^ mice

**Fig. 5:**
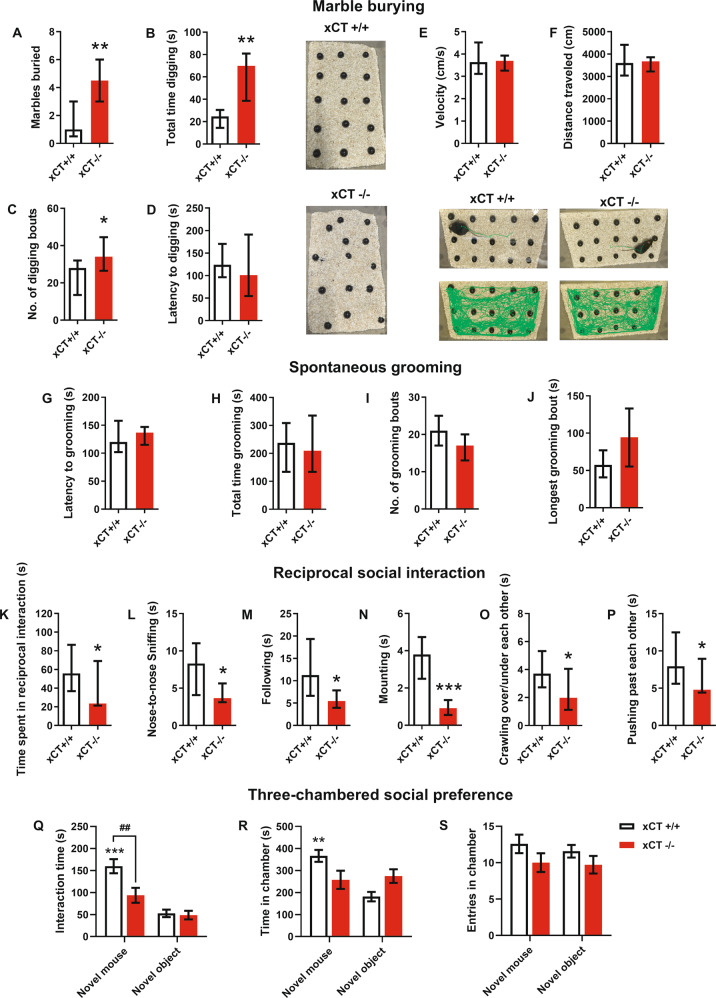
Marble burying, grooming, and social behavior in xCT^−/−^ vs. xCT^+/+^ mice. Genetic deletion of xCT led to a significant increase in the number of marbles buried (**a**), the total time spent digging (**b**) and the total number of digging bouts (**c**) during a 15 min trial, but did not influence the latency to digging (**d**). Right panels show representative images of the distribution and coverage of marbles at the end of the test in xCT^−/−^ and xCT^+/+^ mice. No difference in velocity (**e**) or total distance traveled (**f**) could be detected between genotypes, indicating similar levels of locomotor activity during the test. *n* = 9 xCT^+/+^ mice, *n* = 10 xCT^−/−^ mice. Absence of xCT did not influence grooming behavior as evaluated in a 30 min trial, including latency to grooming (**g**), total time spent grooming (**h**), total number of grooming bouts (**i**), or duration of the longest grooming bout (**j**). *n* = 11 xCT^+/+^ mice, *n* = 11 xCT^−/−^ mice. xCT^−/−^ mice tested in reciprocal social interaction showed overall decreased time engaging in social interaction compared with xCT^+/+^ littermates (**k**). This effect was mainly driven by decreases in time spent in nose-to-nose sniffing (**l**), following (**m**), mounting (**n**), crawling over/under each other (**o**), and pushing past each other (**p**). Other parameters including the time engaging in nose-to-body sniffing, nose-to-anogenital sniffing, allogrooming, chasing, and wrestling/fighting remained unchanged between genotypes (data not shown). *n* = 9 xCT^+/+^ couples, *n* = 6 xCT^−/−^ couples. Data are presented as median ± interquartile range and analyzed using a two-tailed Mann–Whitney *t* test. *p < 0.05, **p < 0.01, ***p < 0.001. **q–s** xCT^−/−^ mice demonstrated no preference to interact with a novel mouse compared with a novel object in the three-chambered social preference test, whereas xCT^+/+^ mice demonstrate intact sociability. The time spent interacting with the novel mouse vs. the novel object (**q**) and the time spent in the chamber containing the novel mouse vs. the novel object (**r**) were significantly higher in xCT^+/+^, but not xCT^−/−^ mice. No differences were observed in the number of entries in the two chambers between the genotypes indicating that loss of xCT does not influence the motivation to explore the assay (**s**). *n* = 7 xCT^+/+^ mice, *n* = 7 xCT^−/−^ mice. Data are presented as mean ± standard error of the mean and analyzed using two-way ANOVA (**q**: genotype × chamber factor: *F*(1,24) = 5.46, *p* < 0.05; **r**: genotype × chamber factor: *F*(1,24) = 10.5, *p* < 0.01) followed by Tukey post hoc test for multiple comparisons (**p < 0.01, ***p < 0.001 Tukey post hoc xCT^+/+^ Novel mouse vs. Novel object, ##*p* < 0.01 Tukey post hoc xCT^+/+^ Novel mouse vs. xCT^−/−^ Novel mouse; (**q**, **r**)). Mice were tested at 12–14 weeks of age for marble burying and spontaneous grooming, and at 8–9 weeks of age for reciprocal social interaction and three-chambered social preference tests.

Phenotypes observed in OCD and autism, such as repetitive behavior and deficits in social interaction, can be correlated to dysregulated corticostriatal transmission [10, 11]. We therefore tested the xCT^−/−^ mice in the marble burying test to evaluate digging perseveration and repetitive behavior. The xCT^−/−^ mice had a higher number of marbles buried at the end of the trial (Fig. 5a), increased time digging (Fig. 5b), and total number of digging bouts (Fig. 5c), with no changes in the latency to digging (Fig. 5d). This was not a consequence of hyperactivity, as the locomotor function of xCT^−/−^ mice during the marble burying test was indistinguishable from that of xCT^+/+^ controls (Fig. 5e, f). No changes in spontaneous grooming behavior could be observed in xCT^−/−^ mice (Fig. 5g–j), suggesting that the stereotypical behavior observed in the marble burying test does not immediately generalize to other forms of repetitive behavior.

In the reciprocal social interaction test, xCT^−/−^ mice demonstrated decreased sociability, with reduced time spent in reciprocal interaction with age-, sex-, and genotype-matched socially novel mice, compared with xCT^+/+^ littermates (Fig. 5k–p). To confirm this pattern of decreased sociability in xCT^−/−^ mice, we employed the three-chambered social preference test. When presented with a choice to explore a socially naive mouse or an inanimate object, xCT^+/+^ mice demonstrated a preference for interacting with the mouse, contrary to xCT^−/−^ mice (Fig. 5q, r). Importantly, the exploratory activity of the two genotypes was similar (Fig. 5s).

## Discussion

Despite its functional contribution to maintaining extracellular glutamate levels in distinct brain regions [2, 3, 57], the function of system x_c_^−^ in regulating synaptic excitatory neurotransmission has remained incompletely understood. Here we investigated the role of system x_c_^−^ in modulating neurotransmission at corticostriatal synapses, one of the two major types of striatal excitatory inputs.

Our findings revealed that glutamate released by system x_c_^−^ is required for physiological corticostriatal neurotransmission. The fEPSPs recorded at the level of the dorsolateral striatum following stimulation of corticostriatal fibers were reduced in xCT^−/−^ compared with xCT^+/+^ mice, while exogenous application of physiological levels of glutamate rescued this deficit. We could not detect changes in the paired-pulse ratios, indicating absence of changes in short-term synaptic plasticity. These results are in agreement with a recent study indicating that inhibition of system x_c_^−^ leads to reduced evoked excitatory postsynaptic currents in prefrontal cortex layer II/III pyramidal neurons, suggesting a functional role of system x_c_^−^ in maintaining excitatory synaptic transmission [7]. Of note, genetic deletion of system x_c_^−^ leads to potentiated postsynaptic responses at hippocampal CA3-CA1 synapses [6], while activation of system x_c_^−^ leads to decreased postsynaptic responses at cortico-accumbens synapses [5], suggesting that the function of system x_c_^−^ may be circuit-dependent.

To better understand the underlying substrate, we investigated the presence of structural and morphological modifications in striatal MSNs of xCT^−/−^ mice. We failed to identify any noticeable effect on the density of spines and corticostriatal synapses, indicating that the decrease in neurotransmission is not due to any significant spine or synapse loss. In addition, the morphology of striatal MSNs and the ultrastructure of corticostriatal synapses were unchanged in xCT^−/−^ mice. Similarly, the synapse density and synapse morphology is preserved at the level of the hippocampus of xCT *sut*/*sut* mice, another system x_c_^−^ loss of function model [58]. However, by combining EM with glutamate immunogold labeling, we uncovered a decrease in glutamate concentrations in various intracellular compartments in xCT^−/−^ mice, including presynaptic terminals, mitochondria, and dendritic spines. Given that xCT^−/−^ mice have normal glutamate content in striatal homogenates reflecting intact synthesis [2], the decrease in glutamate levels may be a result of decreased glutamate in the extracellular environment [2], leading to decreased uptake in terminals and spines. Recent findings indicate that the glutamate reuptake transporter GLT-1 is also present and active in striatal nerve terminals [59], where it could load glutamate inside the presynaptic terminals, which after uptake in synaptic vesicles, can act as an alternative route to the glutamate–glutamine cycle to ensure intracellular glutamate stores [59, 60]. It may be tempting to speculate that the reduced extracellular glutamate levels in the absence of system x_c_^−^ lead to reduced substrate availability for presynaptic glutamate uptake transporters to replenish intracellular glutamate stores during neurotransmission. In turn, the decrease in the presynaptic glutamate stores in the xCT^−/−^ mice may lead to decreased synaptic glutamate release upon stimulation and decreased postsynaptic responses. Importantly, the expression [2] and activity of glutamate transporters in striatal homogenates of xCT^−/−^ mice is preserved (Supplementary Fig. 6), indicating that the decrease in intracellular glutamate is not related to dysfunctional glutamate uptake. Further studies are warranted to better understand the contribution of system x_c_^−^-delivered extracellular glutamate as alternative source for maintaining proper presynaptic glutamate concentrations during neurotransmission.

We took the first steps to expand into the possible molecular mechanisms involved by performing exploratory proteomic and kinomic analyses. Our proteomic findings suggest altered striatal expression of pre- and postsynaptic proteins after loss of xCT. Although small in magnitude, the aggregation of decreased expression of presynaptic proteins including synaptophysin, α-synuclein, and members of the synapsin, septin, and syntaxin families, may contribute to reduced synaptic glutamate release in xCT^−/−^ mice, by impacting synaptic vesicle mobilization, trafficking, docking, and exocytosis. Interference with the presynaptic machinery is supported by the finding that system x_c_^−^ inhibitors sorafenib and erastin induce deficits in presynaptic function in primary hippocampal cultures by modulating presynaptic vesicle docking and decreasing the synaptic vesicle pool size [61]. In addition, kinome array analysis revealed abnormal striatal kinase signaling, with ERK emerging as a possible node of kinase network dysregulation in xCT^−/−^ mice. ERK signaling has been implicated in regulating presynaptic glutamate release via phosphorylation of synapsin-1 [62] and postsynaptic responses by controlling the insertion of synaptic AMPA receptors and AMPA receptor neurotransmission [63], as well as in immediate early gene induction following corticostriatal stimulation in vivo [64]. Both changes in ERK activity [65] and expression of presynaptic proteins such as synapsin-2 [66] and septin-5 [67], have been linked with social interaction and autism-like behaviors, providing a possible pathway connecting system x_c_^−^ deficiency with phenotypes which we observed in vivo.

Indeed, deficits in corticostriatal transmission are associated with psychiatric disorders such as autism and OCD [10, 11]. Typical mouse behavioral phenotypes such as repetitive behavior and disturbed sociability are linked to these human pathologies. The marble burying test revealed increased digging behavior in the absence of system x_c_^−^, suggesting increased repetitive behavior. Yet, absence of pathological grooming behavior does not support the presence of OCD-like behavior. In both paradigms that we used for evaluating social behavior, xCT^−/−^ mice demonstrated decreased preference for social contact compared with their xCT^+/+^ littermates. These findings are consistent with observations that hypofunction of system x_c_^−^ due to regulator of G-protein signaling 4 downregulation leads to decreased synaptic transmission in organotypic slices containing cortex and hippocampus and causes social deficits in vivo [68]. Although social interaction deficits can be induced by corticostriatal dysfunction, it should be noted that dysregulations in glutamatergic neurotransmission in other regions of the brain may contribute to the observed phenotype. In particular, system x_c_^−^ plays an important role in maintaining glutamate homeostasis at the level of the hippocampus [3], and xCT^−/−^ mice demonstrate abnormal hippocampal neurotransmission [6] that may influence social behavior [69].

As increased repetitive behavior and decreased sociability are suggestive of autistic-like behavior, further studies are warranted to investigate a possible implication of system x_c_^−^ hypofunction in autism spectrum disorder and whether stimulating system x_c_^−^ might prove beneficial. Previously, a genome-wide linkage analysis of families with autism identified a susceptibility locus located on chromosome 4 in a region containing SLC7A11, the gene encoding xCT [16]. In addition, a rare missense variation in SLC7A11 has been recently identified in a small family with affected autism spectrum disorder siblings by whole-exome sequencing [15]. Further, N-acetyl-cysteine, a nonspecific system x_c_^−^ activator, reverses social interaction deficits and enhanced presynaptic excitatory neurotransmission at thalamic-amygdala synapses in a valproic acid-induced rat model of autism [17], and decreases marble burying behavior in mice [18].

In conclusion, we provide evidence that system x_c_^−^ regulates corticostriatal neurotransmission in physiological conditions and has functional effects on behavioral phenotypes modulated by this brain circuit, including repetitive behavior and decreased sociability. These changes were not associated with changes in synapse or spine density at the level of the dorsolateral striatum but occurred in the presence of decreased pre- and postsynaptic glutamate concentrations, changes in presynaptic protein expression, and dysregulated kinase signaling. Future studies should help to clarify the relevance of system x_c_^−^ in psychiatric disorders such as autism.

## Supplementary information

Supplementary information
