# Supplementary material for: Corticostriatal dysfunction and social interaction deficits in mice lacking the cystine/glutamate antiporter
Source: Mol Psychiatry. 2020 May 4;26(9):4754–69. doi: 10.1038/s41380-020-0751-3 (PMC7609546; doi:10.1038/s41380-020-0751-3)
Supplement: Supplementary file 1 — Supplementary information [file 41380_2020_751_MOESM1_ESM.docx]

**Supplementary Methods**

**Genotyping**

Genotypes were confirmed by PCR amplification of ear DNA using REDExtract-N-Amp Tissue PCR Kit (Sigma), and the following primers: 5′-GATGCCCTTCAGCTCGATGCGGTTCACCAG-3′ (GFPR3); 5′-CAGAGCAGCCCTAAGGCACTTTCC-3′ [mxCT5′flankF6]; and 5′-CCGATGACGCTGCCGATGATGATGG-3′ [mxCT(Dr4)R8]. The principle of the PCR amplification and an example of a genotyping gel is shown in Supplementary Fig.1.

**Proteomics**

***Nano liquid chromatography coupled to electrospray tandem mass spectrometry***

Nano liquid chromatography coupled to electrospray tandem mass spectrometry (nLC-ESI-MS/MS) analyses were performed on a 5600+ QTOF mass spectrometer (Sciex, Toronto, On, Canada) with an Eksigent (Dublin, CA) nanoLC ultra nanoflow system, as described in detail previously [[1](#_ENREF_1)], but with the following details. First, the samples were prepared such that 2.5 µg of protein digest containing 1X of iRT (indexed Retention Time) internal standard (#Ki-3002-1) as described by the vendor (Biognosys, Boston, USA) was used for each injection. For Data Dependent Acquisition (DDA) each sample was analyzed as technical triplicates. The system was operated in positive ion mode set to 2349 cycles over the 90 min gradient run. Each cycle included 1 TOFMS scan covering m/z 350-1250 with 250 ms accumulation time followed by isolation and fragmentation of the 50 most intense ions with charge states of +2 to +4 with an accumulation time of 50 msec resulting in a full duty cycle of just under 2.75 sec. Former MS/MS-analyzed candidate ions were excluded for 12 sec after its first occurrence to reduce the redundancy of the identified peptides. For Data Independent Acquisition (DIA), each 2.5 µg sample injection was separated using the same 90 min gradient as above and was subjected to a custom series of overlapping mass windows for data collection covering the m/z range of 400-1250 and including a 1 Da overlap of successive windows (Supplementary Table 1). This produced 1700 cycles where each duty cycle included 1 TOFMS scan covering m/z 350-1250 at 200 ms accumulation time followed by 100 DIA segments each with a 28 ms accumulation time for a total duty cycle of 3.05s. The NanoSpray III ion source (Sciex) parameters used of all analyses included a source gas 1 (GS1), source gas 2 (GS2), and curtain gas (CUR) set to 8, 0, 35, respectively. The system was operation in positive ion mode with the heated interface and the ion spray voltage set to 150 C and 2.6kV. MS/MS spectra were all collected using the rolling collision energy function with an energy spread of 15 as provided in the Analyst-TF v. 1.7 (Sciex) data acquisition software.

**Kinomics**

***Sample preparation***

For measuring kinase activity, striatal tissue was lysed on ice for 30min using M-PER lysis buffer (ThermoFisher) containing 1:100 Halt Protease and Phosphatase Inhibitor Cocktail (ThermoFisher). Samples were centrifuged (14000rpm, 10min, 4^o^C) and the supernatants collected. Thirty µg protein from each of respectively the 9 xCT^+/+^ and 10 xCT^−/−^ samples were pooled. Samples were run in duplicate using two separate chips, and the results averaged across the two arrays.

***Serine-threonine kinase activity profiling***

Profiling of serine-threonine kinase (STK) activity was performed using the PamStation12 microarray (PamGene International) and STK PamChips containing 144 consensus phosphopeptide sequences (3 of which are internal controls) per well, immobilized on porous ceramic membranes [[2-4](#_ENREF_2)]. Each PamChip well was blocked with 2% bovine serum albumin (BSA) before adding 2µg of protein in the manufacturer’s kinase buffer (PamGene), 157µM adenosine triphosphate (ATP), and FITC-labeled anti-phospho serine-threonine antibodies (PamGene). The homogenized samples and assay mix were pumped through the wells to facilitate interaction between kinases in the sample and specific peptide substrates immobilized on the chip. The degree of phosphorylation per well was measured in real time using Evolve (PamGene) kinetic image capture software, capturing FITC-labeled anti-phospho antibodies binding to each phosphorylated peptide substrate every 6sec for 60min. Peptide spot intensity was captured across multiple exposure times (10, 20, 50, 100, 200ms) during post-wash, and the linear regression slope was calculated and used as the signal (i.e. peptide phosphorylation intensity) in comparative analyses. The signal ratio between xCT^+/+^ and xCT^−/−^ samples was used to calculate fold change (FC) for each peptide. Peptides with a FC of at least 10% (i.e. FC > 1.10 or FC < 0.90) were considered as different in degree of phosphorylation [[3](#_ENREF_3), [5](#_ENREF_5)]. Peptides that were undetectable or non-linear in the post-wash phase (i.e. the coefficient of determination R^2^ of the corresponding linear regression less than 0.90) were excluded from subsequent analyses. From the 141 peptides, 66 peptides were excluded under this criterion. In addition, a control array was run without the addition of ATP to identify nonspecific binding of the labeled antibody to the array substrate. Peptides with significant background signal in the −ATP condition (higher than the +ATP condition) were excluded from subsequent analyses. Seven peptide probes were excluded based on this criterion (Supplementary Fig.4), leaving a total of 70 substrates in the final analysis.

***Waterfall plots and individual peptide phosphorylation curves***

For comparing phosphorylation levels between xCT^−/−^ and xCT^+/+^ samples, FC values were calculated at individual reporter peptides and averaged across the two replicate chips. A graphical representation of the change in phosphorylation across reporter substrates is illustrated using waterfall plots, in which the reporter peptides are arranged on the y-axis in decreasing order of FC. In addition, post-wash phosphorylation curves are plotted at individual reporter peptides, using the peptide spot intensities captured during post-wash (10, 20, 50, 100, 200ms exposure times), averaged across the two replicate chips.

***Kinase resampling analysis***

Using GPS 3.0 [[6](#_ENREF_6)] and Kinexus Phosphonet (Kinexus Bioinformatics) (http://www.phosphonet.ca), we identified protein kinases acting on phosphorylation sites within the array peptide sequences. These programs provide ranked predictions for serine-threonine kinases targeting putative phosphorylation sites in the peptide sequence. The top 5 kinases predicted by Kinexus and kinases with scores more than twice the prediction threshold for each phosphorylation site in GPS 3.0 were included as predicted kinases for each substrate. This list was complemented by kinases documented to act on the phosphorylation sites of the substrates extracted from public databases PhosphoELM (http://phospho.elm.eu.org), and PhosphoSite Plus (https://www.phosphosite.org). To determine which upstream kinases are most likely to be important in the xCT^−/−^ signaling network, we performed random sampling analysis [[2](#_ENREF_2), [3](#_ENREF_3)]. We generated data sets (n=2000) where each data point includes randomly selected reporter peptide substrates from the kinome array. Each analysis included 39 substrates, matching the number of substrates significantly changing in xCT^−/−^ mice. We identified kinases predicted to target each phosphorylation site and calculated the frequency of each kinase for all 2000 data points in the data sets. From these we generated an expected distribution for each kinase. Means and standard deviations were calculated for each expected distribution. Kinases with observed frequencies falling outside two standard deviations from the expected mean (derived from randomly generated data points) were carried forward into our network analyses.

***Kinase network modeling***

Kinases implicated by the random sampling analyses were used to create a kinase signaling network. We generated a network model that represents the number of direct interactions between protein kinases identified from our random sampling analyses, as well as additional protein kinases that are known to be connected with our array hits. For growing and connecting the kinase network we used the Search Tool for Retrieval of Interacting Genes/Proteins (STRING) database (http://string-db.org), selecting interactors that connect with the kinase proteins using a high confidence interaction score. Because signaling networks may be amplified based on the number of interactions between kinases, we weighted our model based on the number of interactions found for each kinase in the network. Visualization of the obtained kinase network model was performed using Cytoscape ver. 3.6.1 [[7](#_ENREF_7)].

***Exploratory kinome array studies***

For investigating the role of extracellular signal-regulated kinase (ERK) in regulating the kinase network in xCT^−/−^ and xCT^+/+^ mice, each sample was evaluated with the kinome array in the presence or absence of a specific ERK1/2 inhibitor (FR 180204; Tocris 3706), added at a concentration of 5µM [[8](#_ENREF_8)]. The ratio of kinase activity in the inhibitor / no inhibitor samples for each peptide substrate was used to calculate FC data. Differences in FC were calculated as: [(xCT^−/−^ with inhibitor) / (xCT^−/−^ without inhibitor)] – [(xCT^+/+^ with inhibitor) / (xCT^+/+^ without inhibitor)]. For generating the peptide phosphorylation heatmap and comparing the global phosphorylation levels between four different groups (xCT^+/+^ with inhibitor, xCT^+/+^ without inhibitor, xCT^−/−^ with inhibitor, xCT^−/−^ without inhibitor), the linear regression slope of each peptide was multiplied by 100 and log2 transformed [[2](#_ENREF_2), [3](#_ENREF_3)]. The peptide phosphorylation heatmap was generated in R Software (R version 3.4.2, 2017 The R Foundation for Statistical Computing). For ease of representation, the heatmap is normalized per row, to better reflect relative changes in phosphorylation at individual peptides between the four groups.

**Western blotting**

Samples were prepared for Western blot analysis, proteins separated on a 4–12% Bis–Tris Invitrogen NuPage gel and transferred to PVDF membranes as described before [[9](#_ENREF_9)]. Membranes blocked for 1h at room temperature using LiCor Odyssey blocking buffer (LiCor, Lincoln, NE, USA), before overnight incubation at 4°C in primary antibody solution: mouse p44/42 MAPK (ERK1/2) (1:250, Cell Signaling 4696), rabbit phospho-p44/42 (p-ERK1/2) (1:250, Cell Signaling 4370), and rabbit synaptophysin (1:2000, Cell Signaling 5467S). After incubation with donkey anti-mouse IR-Dye 670 labeled secondary antibody or goat anti-rabbit IR-Dye 800cw labeled secondary antibody (both 1:5000, LiCor), membranes were scanned (LiCor Odyssey scanner). The intensity value for each protein band was measured using the Odyssey 3.0 software and normalized to the total protein amount obtained by Ponceau staining.

**Measurment of D-[^3^H]-aspartate uptake in striatal synaptosomes**

Glutamate uptake activity was measured in freshly prepared striatal synaptosomes from xCT^−/−^ and xCT^+/+^ mice using D-[^3^H]-aspartate as substrate at a tracer concentration of 50nM, as described previously [[10](#_ENREF_10)]. The relative involvement of the two main glutamate transporter subtypes was analyzed by measuring D-[^3^H]-aspartate uptake in the presence and absence of specific GLAST (UCPH101 10μM) or GLT-1 (WAY213613 5μM) inhibitors. Non-specific uptake was measured in the presence of the non-selective glutamate uptake inhibitor L-(-)-threo-3-hydroxyaspartic acid (LTHA) at a concentration of 500µM.

**Supplementary Figures**


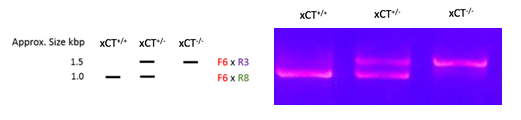


**Supplementary Fig.1.** Genotyping principle and example of genotyping gel allowing identification of wild-type, heterozygous, and homozygous knock-out mice. The primer GFPR3 is designated as R3, mxCT5′flankF6 as F6 and mxCT(Dr4)R8 as R8.

**
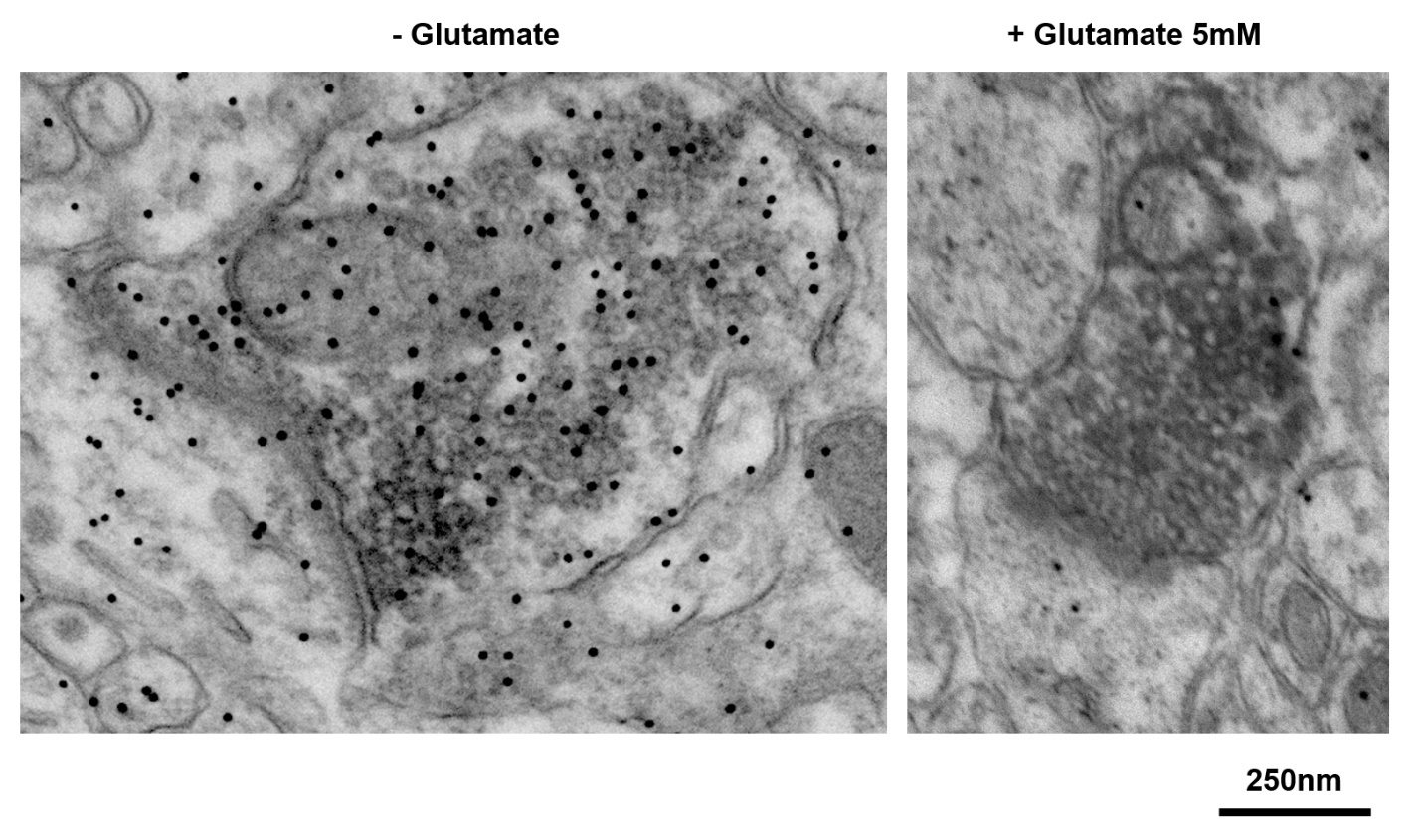
Supplementary Fig.2.** Specific glutamate immuno-gold labeling in the conditions utilized in the current study. The primary glutamate antibody was incubated overnight with 5 mM glutamate (right picture, + Glutamate 5mM) and then applied to thin sectioned striatal tissue that was pre-embed immuno-labeled for VGLUT1. In comparison with tissue incubated only with the primary antibody (left picture, - Glutamate), the addition of glutamate 5mM effectively competed out the binding to glutamate present in the tissue, demonstrating the specificity of binding.

**
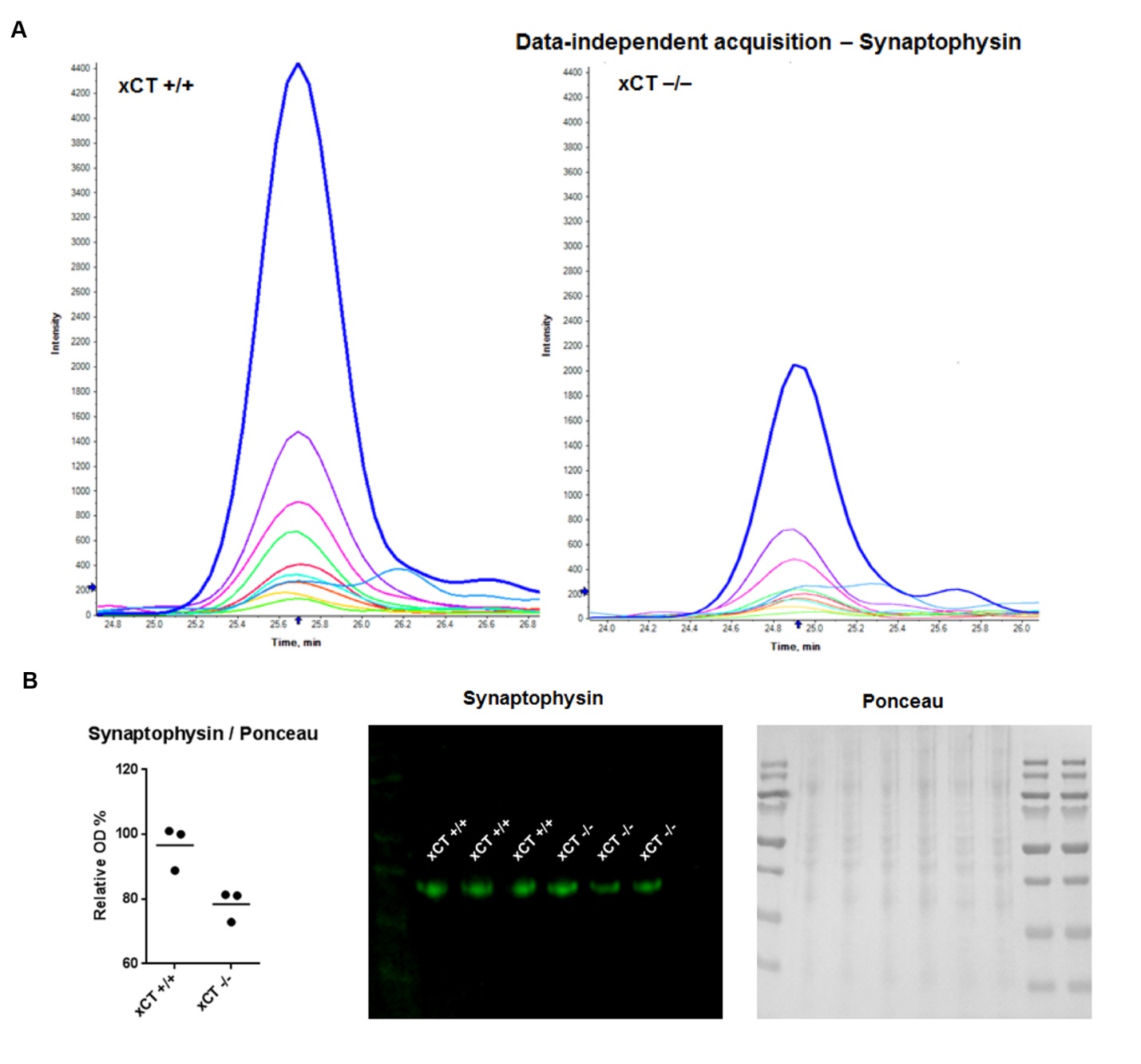
**

**Supplementary Fig.3.** Proteomic analysis of xCT^−/−^ and xCT^+/+^ mice. By running proteomics in data-independent acquisition (DIA) mode, and performing Western blot using the same samples analyzed for proteomics, we identified a similar decrease in synaptophysin expression in xCT^−/−^ samples as observed during DDA (Fig. 4A). (A) Chromatogram of synaptophysin fragmentation spectra in xCT^−/−^ and xCT^+/+^ samples obtained during proteome analysis run in DIA mode. (B) Western blot analysis of synaptophysin in xCT^+/+^ samples (pooled from n=9 biological replicates) and xCT^−/−^ samples (pooled from n=10 biological replicates). Samples were loaded in technical triplicate on a 12% Bis–Tris Invitrogen NuPage gel, transferred to a PVDF membrane, and probed with synaptophysin antibody. Normalization was performed using Ponceau total protein stain on the same membrane.


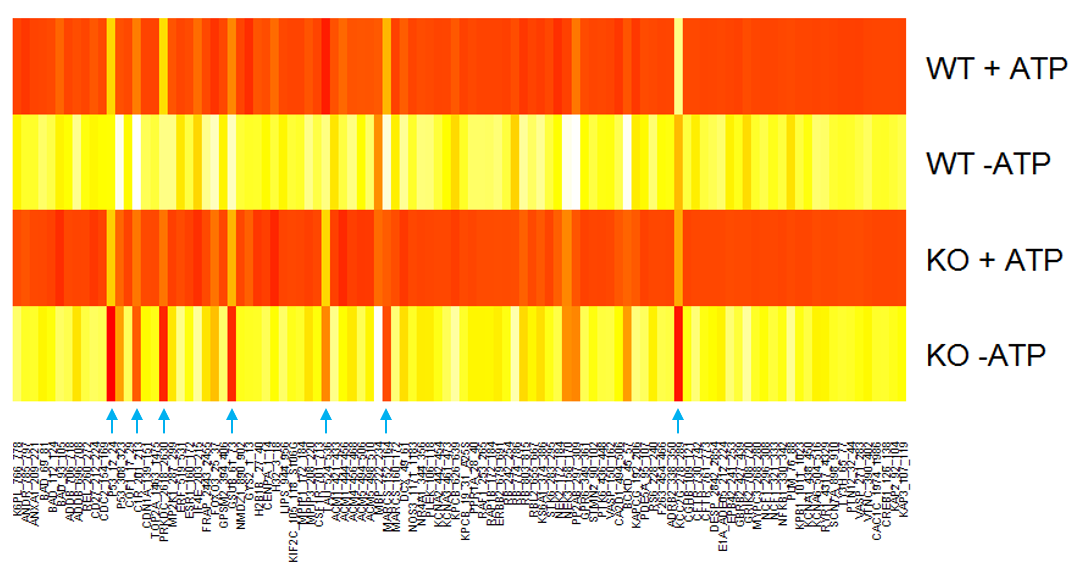


**Supplementary Fig.4.** Specificity of signal obtained using the PamGene serine/threonine kinase array on striatal tissue of xCT^+/+^ and xCT^−/−^ mice. Nonspecific signal (higher in the –ATP condition than +ATP condition) was detected on 7 peptide substrates (indicated by blue arrow), which were excluded from subsequent analyses. For ease of clarity, the heatmap is normalized per row to highlight relative changes at each individual peptide between the groups. Orange-red indicates high levels of phosphorylation and yellow-white indicates low levels of phosphorylation.

**
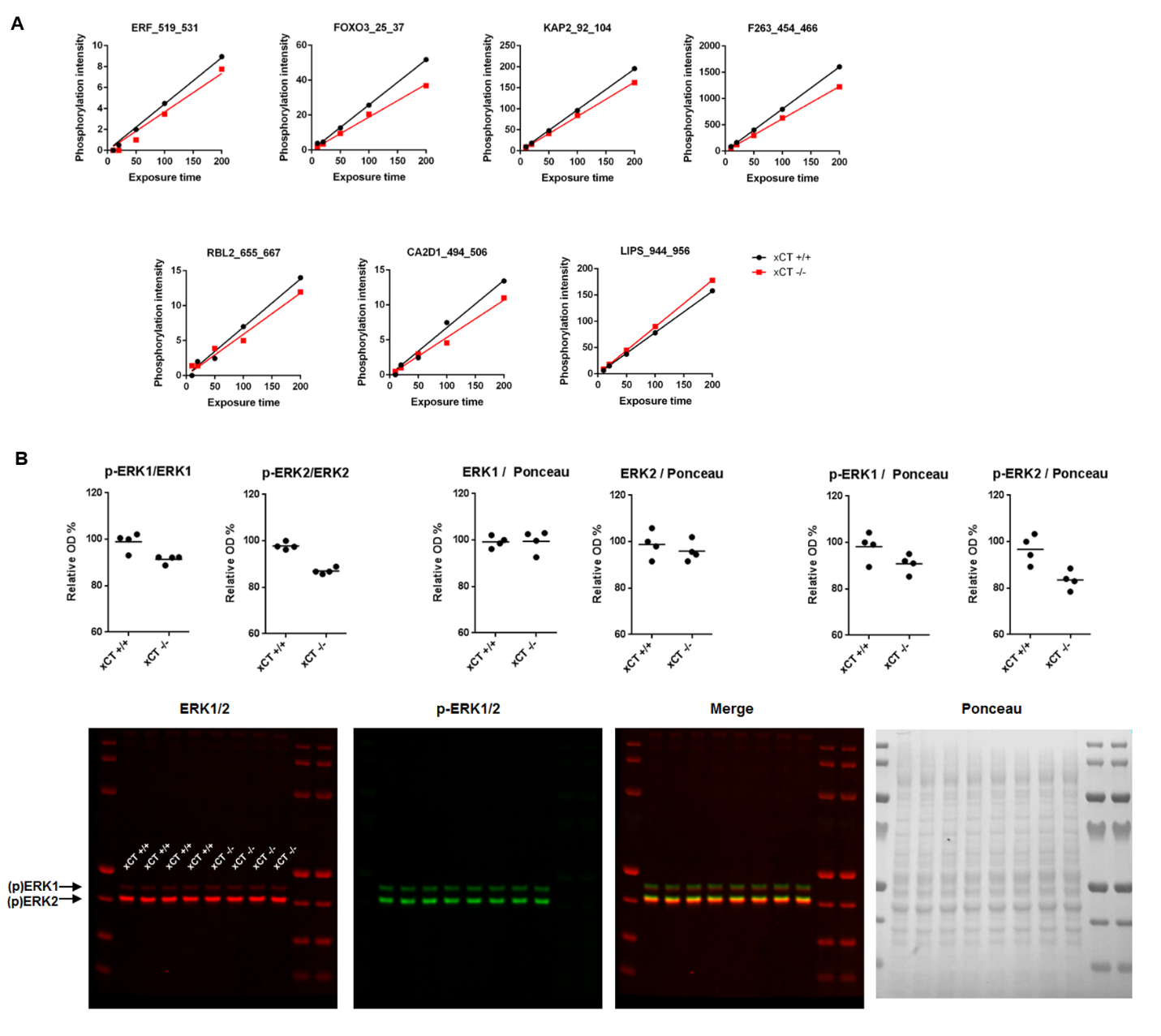
**

**Supplementary Fig.5.** (A) Representative examples of post-wash phosphorylation curves at seven ERK-sensitive reporter peptides in xCT^−/−^ vs xCT^+/+^ mice. (B) ERK1/2 and p-ERK1/2 expression levels in the striatum of xCT^−/−^ mice. Western blot analysis of the samples subjected to kinome analysis, revealed decreased levels p-ERK1/ERK1 and p-ERK2/ERK2 in the striatum of xCT^−/−^ mice, indicating decreased ERK1/2 activation. xCT^+/+^ samples (pooled from n=9 biological replicates) and xCT^−/−^ samples (pooled from n=10 biological replicates) were loaded in technical quadruplicate on a 4-12% Bis–Tris Invitrogen NuPage gel, transferred to PVDF membrane, and probed with ERK1/2 and p-ERK1/2 antibodies. Ponceau total protein stain of the same membrane was used to control for equal amounts of protein loaded.


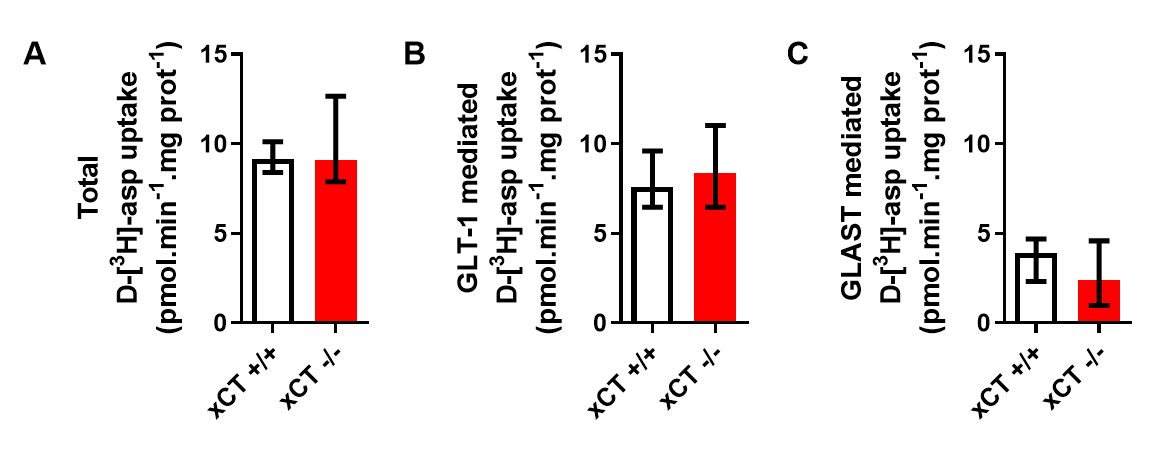


**Supplementary Fig.6.** D-[^3^H]-aspartate uptake in striatal synaptosomes of xCT^−/−^ and xCT^+/+^ mice. No difference was observed in total specific aspartate uptake activity (A), GLT-1-mediated uptake activity (B; synaptosomes incubated with specific GLAST inhibitor UCPH101), or GLAST-mediated uptake activity (C; synaptosomes incubated with specific GLT-1 inhibitor WAY213613). Data are presented as median ± interquartile range and analyzed using a two-tailed Mann-Whitney t test.

| **Supplementary Table 1.** DIA variable mass windows method | | | |
| --- | --- | --- | --- |
| 100 DIA segments | Start m/z, Stop m/z | 100 DIA segments | Start m/z, Stop m/z |
| Segment 1 | 400.00, 420.00 | Segment 51 | 699.00, 703.00 |
| Segment 2 | 420.00, 430.00 | Segment 52 | 703.00, 707.00 |
| Segment 3 | 430.00, 440.00 | Segment 53 | 707.00, 711.00 |
| Segment 4 | 440.00, 450.00 | Segment 54 | 711.00, 715.00 |
| Segment 5 | 450.00, 460.00 | Segment 55 | 715.00, 719.00 |
| Segment 6 | 460.00, 470.00 | Segment 56 | 719.00, 723.00 |
| Segment 7 | 470.00, 479.00 | Segment 57 | 723.00, 727.00 |
| Segment 8 | 479.00, 488.00 | Segment 58 | 727.00, 731.00 |
| Segment 9 | 488.00, 497.00 | Segment 59 | 731.00, 735.00 |
| Segment 10 | 497.00, 506.00 | Segment 60 | 735.00, 739.00 |
| Segment 11 | 506.00, 514.00 | Segment 61 | 739.00, 743.00 |
| Segment 12 | 514.00, 522.00 | Segment 62 | 743.00, 747.00 |
| Segment 13 | 522.00, 530.00 | Segment 63 | 747.00, 751.00 |
| Segment 14 | 530.00, 537.00 | Segment 64 | 751.00, 755.00 |
| Segment 15 | 537.00, 544.00 | Segment 65 | 755.00, 759.00 |
| Segment 16 | 544.00, 551.00 | Segment 66 | 759.00, 763.00 |
| Segment 17 | 551.00, 557.00 | Segment 67 | 763.00, 767.00 |
| Segment 18 | 557.00, 563.00 | Segment 68 | 767.00, 771.00 |
| Segment 19 | 563.00, 569.00 | Segment 69 | 771.00, 775.00 |
| Segment 20 | 569.00, 574.00 | Segment 70 | 775.00, 779.00 |
| Segment 21 | 574.00, 579.00 | Segment 71 | 779.00, 783.00 |
| Segment 22 | 579.00, 584.00 | Segment 72 | 783.00, 787.00 |
| Segment 23 | 584.00, 589.00 | Segment 73 | 787.00, 791.00 |
| Segment 24 | 589.00, 594.00 | Segment 74 | 791.00, 795.00 |
| Segment 25 | 594.00, 599.00 | Segment 75 | 795.00, 799.00 |
| Segment 26 | 599.00, 603.00 | Segment 76 | 799.00, 804.00 |
| Segment 27 | 603.00, 607.00 | Segment 77 | 804.00, 809.00 |
| Segment 28 | 607.00, 611.00 | Segment 78 | 809.00, 814.00 |
| Segment 29 | 611.00, 615.00 | Segment 79 | 814.00, 819.00 |
| Segment 30 | 615.00, 619.00 | Segment 80 | 819.00, 824.00 |
| Segment 31 | 619.00, 623.00 | Segment 81 | 824.00, 829.00 |
| Segment 32 | 623.00, 627.00 | Segment 82 | 829.00, 835.00 |
| Segment 33 | 627.00, 631.00 | Segment 83 | 835.00, 841.00 |
| Segment 34 | 631.00, 635.00 | Segment 84 | 841.00, 847.00 |
| Segment 35 | 635.00, 639.00 | Segment 85 | 847.00, 853.00 |
| Segment 36 | 639.00, 643.00 | Segment 86 | 853.00, 859.00 |
| Segment 37 | 643.00, 647.00 | Segment 87 | 859.00, 865.00 |
| Segment 38 | 647.00, 651.00 | Segment 88 | 865.00, 872.00 |
| Segment 39 | 651.00, 655.00 | Segment 89 | 872.00, 879.00 |
| Segment 40 | 655.00, 659.00 | Segment 90 | 879.00, 886.00 |
| Segment 41 | 659.00, 663.00 | Segment 91 | 886.00, 893.00 |
| Segment 42 | 663.00, 667.00 | Segment 92 | 893.00, 900.00 |
| Segment 43 | 667.00, 671.00 | Segment 93 | 900.00, 907.00 |
| Segment 44 | 671.00, 675.00 | Segment 94 | 907.00, 915.00 |
| Segment 45 | 675.00, 679.00 | Segment 95 | 915.00, 923.00 |
| Segment 46 | 679.00, 683.00 | Segment 96 | 923.00, 932.00 |
| Segment 47 | 683.00, 687.00 | Segment 97 | 932.00, 941.00 |
| Segment 48 | 687.00, 691.00 | Segment 98 | 941.00, 961.00 |
| Segment 49 | 691.00, 695.00 | Segment 99 | 961.00, 1011.00 |
| Segment 50 | 695.00, 699.00 | Segment 100 | 1011.00, 1250.00 |

**Supplementary References**

1. Chutipongtanate S, Greis KD. Multiplex Biomarker Screening Assay for Urinary Extracellular Vesicles Study: A Targeted Label-Free Proteomic Approach. Sci Rep. 2018;8(1):15039.

2. Bentea E, Depasquale EAK, O'Donovan SM, Sullivan CR, Simmons M, Meador-Woodruff JH, et al. Kinase network dysregulation in a human induced pluripotent stem cell model of DISC1 schizophrenia. Mol Omics. 2019;15(3):173-88.

3. McGuire JL, Depasquale EA, Funk AJ, O'Donnovan SM, Hasselfeld K, Marwaha S, et al. Abnormalities of signal transduction networks in chronic schizophrenia. NPJ Schizophr. 2017;3(1):30.

4. McGuire JL, Hammond JH, Yates SD, Chen D, Haroutunian V, Meador-Woodruff JH, et al. Altered serine/threonine kinase activity in schizophrenia. Brain Res. 2014;1568:42-54.

5. Appuhamy JA, Nayananjalie WA, England EM, Gerrard DE, Akers RM, Hanigan MD. Effects of AMP-activated protein kinase (AMPK) signaling and essential amino acids on mammalian target of rapamycin (mTOR) signaling and protein synthesis rates in mammary cells. J Dairy Sci. 2014;97(1):419-29.

6. Xue Y, Liu Z, Gao X, Jin C, Wen L, Yao X, et al. GPS-SNO: computational prediction of protein S-nitrosylation sites with a modified GPS algorithm. PLoS One. 2010;5(6):e11290.

7. Cline MS, Smoot M, Cerami E, Kuchinsky A, Landys N, Workman C, et al. Integration of biological networks and gene expression data using Cytoscape. Nat Protoc. 2007;2(10):2366-82.

8. Ohori M, Kinoshita T, Okubo M, Sato K, Yamazaki A, Arakawa H, et al. Identification of a selective ERK inhibitor and structural determination of the inhibitor-ERK2 complex. Biochem Biophys Res Commun. 2005;336(1):357-63.

9. Sullivan CR, Funk AJ, Shan D, Haroutunian V, McCullumsmith RE. Decreased chloride channel expression in the dorsolateral prefrontal cortex in schizophrenia. PLoS One. 2015;10(3):e0123158.

10. Massie A, Goursaud S, Schallier A, Vermoesen K, Meshul CK, Hermans E, et al. Time-dependent changes in GLT-1 functioning in striatum of hemi-Parkinson rats. Neurochem Int. 2010;57(5):572-8.
